# A 3D structural model and dynamics of hepatitis C virus NS3/4A protease (genotype 4a, strain ED43) suggest conformational instability of the catalytic triad: implications in catalysis and drug resistivity

**DOI:** 10.1080/07391102.2013.800001

**Published:** 2013-06-14

**Authors:** Bradley Rimmert, Salwa Sabet, Edward Ackad, Mohammad S. Yousef

**Affiliations:** a Department of Physics, College of Arts and Sciences, Southern Illinois University Edwardsville, Edwardsville, IL 62026-1654, USA; b Department of Zoology, Faculty of Science, Cairo University, Giza 12613, Egypt; c Biophysics Department, Faculty of Science, Cairo University, Giza, 12613, Egypt

**Keywords:** HCV, catalysis, structure, dynamics, genotype 4

## Abstract

Egypt has the highest prevalence of hepatitis C virus (HCV) infection worldwide with a frequency of 15%. More than 90% of these infections are due to genotype 4, and the subtype 4a (HCV-4a) predominates. Moreover, due to the increased mobility of people, HCV-4a has recently spread to several European countries. The protease domain of the HCV nonstructural protein 3 (NS3) has been targeted for inhibition by several drugs. This approach has had marked success in inhibiting genotype 1 (HCV-1), the predominant genotype in the USA, Europe, and Japan. However, HCV-4a was found to resist inhibition by a number of these drugs, and little progress has been made to understand the structural basis of its drug resistivity. As a step forward, we sequenced the NS3 HCV-4a protease gene (strain ED43) and subsequently built a 3D structural model threaded through a template crystal structure of HCV-1b NS3 protease. The model protease, HCV-4a, shares 83% sequence identity with the template protease, HCV-1b, and has nearly identical rigid structural features. Molecular dynamics simulations predict similar overall dynamics of the two proteases. However, local dynamics and 4D analysis of the interactions between the catalytic triad residues (His57, Asp81, and Ser139) indicate conformational instability of the catalytic site in HCV-4a NS3 protease. These results suggest that the divergent dynamics behavior, more than the rigid structure, could be related to the altered catalytic activity and drug resistivity seen in HCV-4a.

## Introduction

Hepatitis C virus (HCV) is a global health concern. Chronic infection of HCV is a common and leading cause for both cirrhosis and hepatocellular carcinoma (Andrade et al., 2009). Approximately 3% of the world's population, or roughly 170 million people, are currently affected by this disease (Levanchy, 2009). Comparatively, the frequency within Egypt is much higher with 15%, or nearly 13 million people, testing HCV seropositive (Miller & Abu-Raddad, 2010). Roughly 90% of the aforementioned Egyptians are carriers of HCV genotype 4, and the subtype 4a (HCV-4a) predominates (Khattab et al., 2011; Nguyen & Keeffe, 2005).

Genotype 1 is the common variant of HCV throughout the USA, Europe, and Japan, and has thus become the focus of much interest and research (Ali, Ahmed, & Idrees, 2010). While no vaccine is available and current therapies had met with limited success, the management and treatment of infections arising from genotype 1 has advanced considerably (Chatel-Chaix, Baril, & Lamarre 2010; Kwo & Vinayek, 2011). In contrast, however, genotype 4 has not undergone adequate scrutiny and as a result, the targeted drug development has stagnated (Kamal & Nasser, 2008).

Due to its importance in the replication cycle of HCV, the serine protease domain of nonstructural protein 3 (NS3) has been an attractive target for the development of effective inhibitors (Heintges, Enche, Putlitz, & Wands, 2001). The NS3 protease cleaves four downstream sites in the polyprotein and is characterized as a serine protease with a chymotrypsin-like fold, which is activated by the NS4A cofactor (Du, Hou, Guan, Tong, & Wang, 2002). Similar to chymotrypsin, the catalytic triad of HCV NS3 protease is comprised of the three essential residues, histidine, aspartic acid, and serine, numbered from the N-terminus of NS3, 57, 81, and 139, respectively (Lin, 2006).

Together, these three residues carry out general acid–base catalysis on target peptides. Throughout the catalytic mechanism, two tetrahedral intermediates are formed. The serine performs a nucleophilic attack on a carbonyl of the substrate and the histidine fulfills the crucial roles as both acid and base that allow the catalysis to progress (Hedstrom, 2002). The aspartic acid stabilizes histidine via hydrogen bonding that also raises the pKa value of the histidine which is essential in catalysis (Fersht & Sperling, 1973; Hedstrom, 2002).

Other interactions have also been shown to affect catalysis. Zinc plays an important role in the structural stability of NS3 protease by enthalpically disfavoring protein denaturation (Abian, Vega, Neira, & Valazquez-Campoy, 2010). Additionally, a bound peptide cofactor (NS4A) increases the protease activity by nearly 1000-fold (Sardana, Blue, Zugay-Murphy, Sardana, & Kuo, 1999).

A several-fold decrease in the catalytic efficiency of HCV-4a NS3 protease has been reported relative to that of HCV-1b (Franco, Clotet, & Martinez, 2008). Several NS3 protease inhibitors, which were designed to interfere with the catalytic triad (e.g. Telaprevir and Boceprevir) have shown promising results with genotype 1, but not with genotype 4 (Chatel-Chaix et al., 2010; Njoroge, Chen, Shih, & Piwinski 2008; White et al., 2010). Very little has been reported on the basis of HCV-4's drug resistance, mainly due to the lack of available 3D structural information.

Molecular dynamics simulations have been useful in the study of the NS3 protease domain of HCV. These simulations elucidated the interaction between the NS4A cofactor and the NS3 protease of genotype 1b (Zhu & Briggs, 2011). They have also granted an insight into the effect of R155 K, A156 V, and D168A mutations on the resistance of HCV-1b to the protease inhibitors, ITMN-191 and TMC435 (Pan, Xue, Zhang, Liu, & Yao, 2012; Xue, Pan, Yang, Liu, & Yao, 2012).

In the current study, we resequenced the gene coding for the HCV-4a (strain ED43) NS3 protease domain and subsequently built a novel 3D structural model including a bound NS4A cofactor and a zinc ion. The model was threaded through the X-ray crystal structure of HCV-1b protease (PDB: 1dy8, Di Marco et al., 2000). We performed molecular dynamics simulations, on both the threading model and the template structure, to investigate the effect of the sequence variability on their respective dynamic behaviors.

Our modeling and molecular dynamics simulations results show that both proteases share very similar rigid and overall dynamic features. Conversely, both proteases exhibit significantly different local dynamics and distance distribution profiles, both in peak values and broadness, at the catalytic triad. Our data suggest that genotype-based structural dynamics could play a significant role in the stability of the catalytic triad, and thereby, in drug response among the HCV genotypes.

## Materials and methods

### DNA sequencing

Isolate ED43 of the HCV-4a genome, inserted in PUC19 vector, was kindly provided by the lab of Dr. Richard M. Elliott (University of Glasgow, Glasgow, Scotland, UK). The complete nucleotide sequence of HCV-ED43, which is 9355 nucleotides long, is deposited in the EMBL database under accession number Y11604 (Chamberlain, Adams, Saeed, Simmonds, & Elliott, 1997). The DNA sequence coding for the NS3 proteins, including the protease, was cloned into pQE-30 vector using pQE-30 primers type III/IV as previously described (Sabet, Al-Sherbiny, Ibrahim, & Hagan, 2009).

To confirm the sequence results, both the template and cloned DNA were sequenced. The two sequencings were done at MBSU DNA sequencing services at the Institute of Biomedical and Life Sciences, University of Glasgow, Scotland, UK. These sequencings were performed using automated sequencer, ABI PRISM model 377 version 3.3.1. The results obtained from the two sequences were aligned to the original ED43 published sequence Y11604. Three sites T54, I134, and R150 in the published sequence were found to be I54, T134, and A150, respectively. The gene coding for the NS4A cofactor was sequenced separately using ABI PRISM model 3730XL analyzer and the result was identical to the published sequence Y11604.

### 3D structure prediction and validation

The 3D structure of HCV-4a NS3 protease was predicted by threading its amino acid sequence through the X-ray crystal structure of HCV-1b NS3 protease(1dy8) via the threading program LOOPP (Di Marco et al., 2000; Meller & Elber, 2001). LOOPP is a fold recognition program that generates atomic coordinates of a sample molecule based on an alignment with a homologous template structure. By integrating the results from direct sequence alignment, sequence profile, threading, secondary structure, and exposed surface area prediction, the LOOPP builds main-chain and all-atom models. A nearly identical model (RMSD 0.156 Å) was also obtained via homology modeling using the SWISS-MODEL Workspace (Arnold, Bordoli, Kopp, & Schwede, 2006; Guex, & Peitsch, 1997; Kiefer, Arnold, Kunzli, Bordoli, & Schwede, 2009; Peitsch, 1995; Schwede, Kopp, Guex, & Peitsch, 2003).

To build the NS4A cofactor, we superposed the model structure onto the template structure (1dy8, RMSD 0.3 Å) and built the sequence of the NS4A cofactor for the model based on the corresponding coordinates found in the template crystal structure. Similarly, a single zinc ion was manually docked at the cysteine triad C97, C99, and C145 into the model guided by the corresponding position in another structure of the template protein (1dxp) in which zinc is present (Di Marco et al., 2000). With the cofactor and zinc bound, the model was energy minimized using the CCP4 program suite (Potterton, Briggs, Turkenburg, & Dodson, 2003; Winn et el., 2011) and the GROMOS96 program, an implementation of the Swiss-pdb viewer (Van Gunsteren et al., 1996).

The final model was validated using the NIH MBI Laboratory for Structural Genomics and Proteomics Structural Analysis and Verification Server. This server utilizes five programs (Procheck, What_Check, ERRAT, Verify_3D, and Prove) to analyze the stereochemical parameters and the quality of the model (Colovos & Yeates, 1993; Hooft, Vriend, Sander, & Abola, 1996; Laskowski, MacArthur, Moss, & Thorton, 1993; Luthy, Bowie, & Eisenberg, 1992; Pontius, Richelle, & Wodak, 1996).

Additionally, CCP4 programs suite 6.0 was used for the calculation of a Ramachandran plot (Ramachandran, Ramakrishnan, & Sasisekharan, 1963), structure superposition, and rmsd value calculation in addition to the evaluation of the stereochemistry (Winn et al., 2011).

### Molecular dynamics simulation

The molecular dynamics simulation (MD) was performed using NAMD 2.9 under the CHARMM27 force field for proteins (MacKerell et al., 1998; MacKerell, Feig, & Brooks, 2004; Phillips et al., 2005). Initially, the 3D structure was solvated using the solvation tool in VMD (Humphrey, Dalke, & Schulten, 1996). The TIP3P model was used for the water molecules (Jorgensen, Chandrasekhar, Madura, Impey, & Klein, 1983). Lengevin dynamics for all nonhydrogen atoms with a damping coefficient of 1 ps^–1^ was used in maintaining a constant temperature of 310 K throughout the system. A constant pressure of 1 atm was maintained using a Nosé–Hoover Langevin piston with a period of 100 fs and damping timescale of 50 fs (Feller, Zhang, Pastor, & Brooks, 1995).

Periodic boundary conditions were used on a 61 Å cubic box with the long-range electrostatics calculated using the particle-mesh Ewald method with a grid point density of 0.92 Å^–1^. This process ensured that adjacent copies of the protease were never close enough for short-range interaction. A cut-off of 10 Å for van der Waals interactions and a switching distance of 8 Å were found to give convergent results, thus used for production runs. The solvation box was neutralized, using VMD's Autoionize plugin version 1.3, with sodium chloride placed at distances greater than 5 Å from the protease.

A time step of 1 fs was used in order to resolve the hydrogen motion of water. The initial structure was first subjected to two rounds of an 800 cycle conjugate gradient energy minimization followed by 50 ps of MD simulation at 100 K. The system was then heated up in increments of 25 K with 50 ps of MD simulation at each temperature increment until the desired temperature of 310 K was established. The system was then simulated for 30ns. Time frames used for the measurements within the protease were only done for frames where the protease had equilibrated: 6–25 ns. The equilibrium state of the protease was determined by the RMSD of the entire protein's backbone.

Multiple copies of each protease, which included the cofactor and a zinc ion (nonbonded), were run with different initial conditions to ensure that the results were well converged. All RMSD data presented are averaged over for at least three distinct runs. Distance distributions are for a single run that was typical of the set of runs to ensure the clarity of interpretation.

## Results and discussion

The NS3 protease domain of HCV is regarded as a chymotrypsin-like serine protease (Tong, 2002) whose catalytic mechanism is well established (Hedstrom, 2002). In summary, a charge relay system is formed in which the carboxylic group of D81 forms a hydrogen bond with Nδ1 of H57 (Hedstrom, 2002). This event increases the p*K*a of the histidine side chain from 7 to about 12 (Fersht & Sperling, 1973; Lin, Cassidy, & Frey, 1998). Consequently, H57 deprotonates the hydroxyl group of the S139 side chain and a proton shuttles to N∊2 of His57 (Hedstrom, 2002). The Oγ of S139 then nucleophilically attacks the carbonyl carbon of a substrate's scissile bond resulting in the formation of an oxyanion-containing tetrahedral intermediate (Hedstrom, 2002; Hirohara, Bender, & Stark, 1974; Raney, Sharma, Moustafa, & Cameron, 2010; Tong, 2002). At this point, the protonated H57 acts as a general acid assisting in the collapse of the tetrahedral intermediate and the cleavage of the substrate (Hedstrom, 2002; Raney et al., 2010). Given the function of the NS3 protease within the viral replication process, the catalytic triad has become an attractive target for rational drug design.

**Figure 1.**
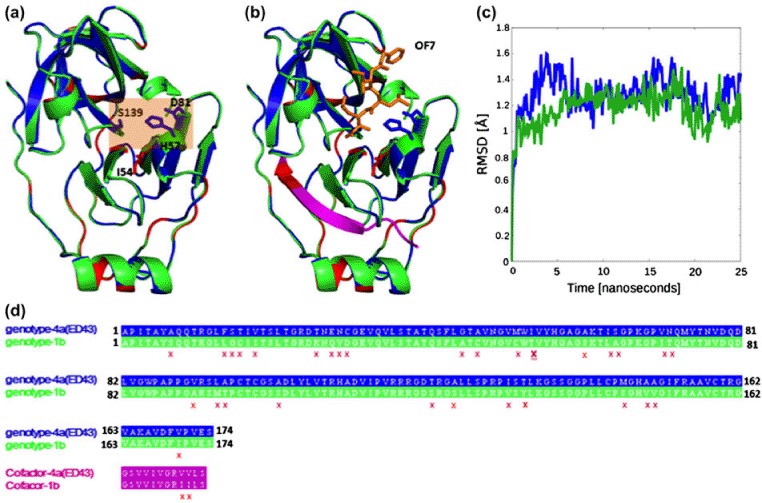
Comparison between the threading model of HCV-4a and the crystal structure of HCV-1b NS3 proteases. (a) The model structure of HCV-4a, shown in blue, is superimposed onto the template structure of HCV-1b (PDB: 1dy8), shown in green. Sequence variability sites are shown in red. The tan box highlights the catalytic triad (H57, D81 and S139). A nearby mutation T54I is shown in red sticks. (b) Same as (a) with the addition of the cofactor (NS4A), in pink, and an example inhibitor shown for orientation purposes (OF7, 1dy8), in orange. (c) Residue-averaged RMSD of Cα atoms for HCV-4a protease model (blue) and HCV-1b protease crystal structure (green) during the course of simulation. (d) The amino acid sequences of HCV-4a and HCV-1b NS3 proteases, as well as the cofactor are shown with the same colors used in (a) and (b). Sequence variability is indicated with a red “X”. The position of T54I is highlighted by a bold underlined red “X”.

The HCV-4a NS3 protease structure model superposes very well on the threading template structure (1dy8), and the two share 83% sequence identity (Figure 1(d)) along with nearly identical structural features (Figure 1(a)). RMSD in back-bone positions between the two proteases is about 0.3 Å. None of the 174-threaded amino acids falls within the disallowed Ramachandran area, and no steric clashes or stereochemical outliers was detected (see Materials and Methods).

**Figure 2.**
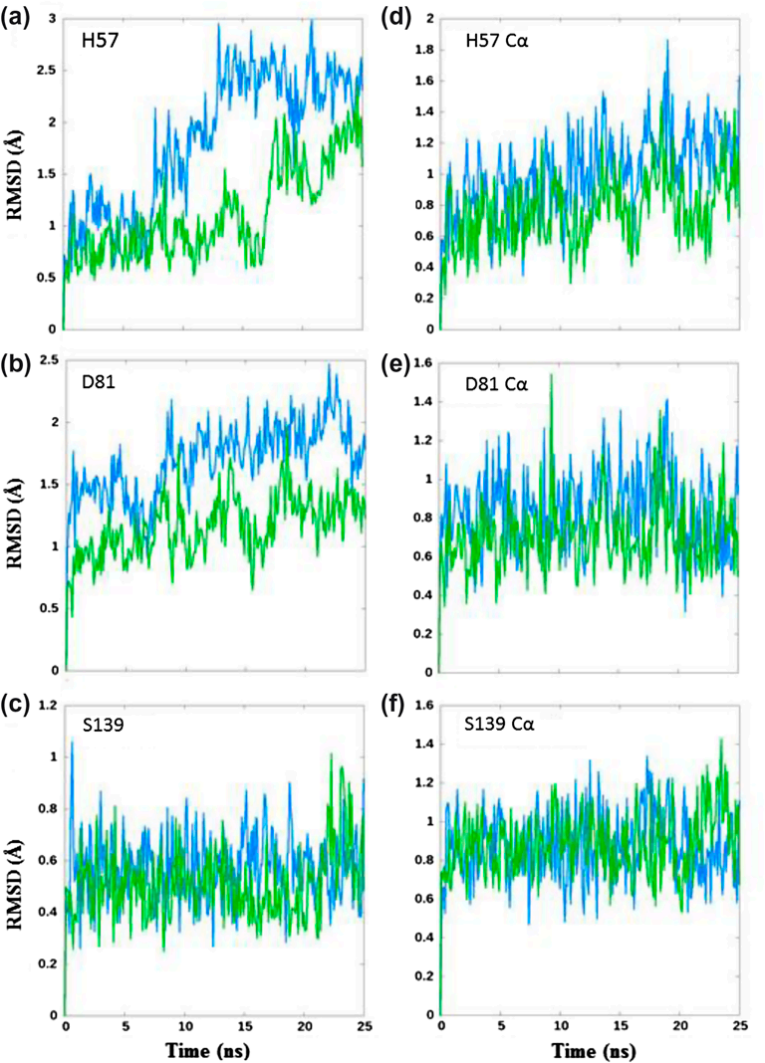
Comparison of the dynamics behavior of the catalytic triad residues between the threading model of HCV-4a (blue) and the crystal structure of HCV-1b (green) proteases. RMSD values for each catalytic residue are shown for the entire residue (a, b, c) and the corresponding alpha carbons (d, e, f).

**Figure 3.**
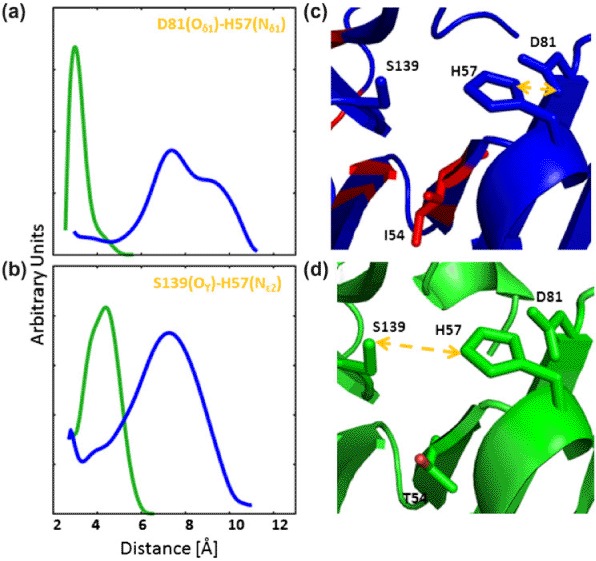
Dynamics behavior within the catalytic triad site of the threading model (HCV-4a, blue) and the template (HCV-1b, green) proteases. The distance distribution profiles between Oδ1 of residues D81 and Nδ1 of H57 (a) and between Oγ of residue S139 and N∊2 of residue H57 (b), during the course of simulation. Orange arrows indicate the selected distances in the rigid structures of both the model (c) and the template (d). Residues shown in red indicate sequence variability.

The three catalytic residues H57, D81, and S139 are located in a crevice between the two protease β-barrels as shown in Figure 1(a) (Barbato et al., 1999; McCoy, Senior, Gesell, Ramanathan, & Wyss, 2001; Yan et al., 1998). The active site is nonpolar and shallow (Hedstrom, 2002). The central region of NS4A is buried almost completely inside the NS3 protease and serves as a cofactor for proper folding of the protease (Figure 1(b)) (Barbato et al., 1999). The rigid structures indicate that access to the active site is nearly identical in both the model and template (Figure 1(a) and (b)). In addition, molecular dynamics simulations predict that both HCV-4a and HCV-1b proteases share similar average RMSD in the Cα positions, ∼1.3 Å at equilibrium (Figure 1(c)). Locally, molecular dynamics simulations revealed divergent dynamics behavior and distance distribution profiles within the catalytic triad region between HCV-4a and HCV-1b proteases (Figures 2 and 3). These dynamic differences are likely to contribute to the altered activity and drug resistivity seen in HCV-4a.

### The dynamical behavior within the catalytic triad

The alpha carbons (Cα) of the catalytic residues exhibit similar dynamics throughout the simulations with those of the HCV-4a model showing slightly higher RMSD, especially for H57 and D81 (Figure 2(d–f)). The S139 side chain also presents a very similar dynamic behavior in both the model (HCV-4a) and template (HCV-1b) (Figure 2(c)). These data are expected since the behavior of Cα atoms, as part of the peptide backbone, would conform to the dynamics seen in Figure 1(c). In addition, the serine presents a relatively small side chain with a movement sterically encumbered by the nearby residue.

However, in the HCV-4a model, H57 and D81 (as entire residues) demonstrate dynamics behavior divergent from that of the template HCV-1b (Figure 2(a) and (b)). The RMSD of H57 in the HCV-4a model varies from that predicted in the HCV-1b template by up to 1.5 Å at certain points during the simulation (Figure 2(a)). Likewise, the RMSD of D81 in the HCV-4a model diverges from that of HCV-1b template by nearly 1 Å (Figure 2(b)). It is possible that the instability of D81, itself fulfilling a stabilizing role for H57, may have deleterious consequences upon the ability of H57 to adequately function as an acid–base. Given the crucial role of H57 in the proteolytic mechanism, our data provide a likely explanation for the reported reduced efficiency of the HCV-4a NS3 protease compared with that of HCV-1b (Franco et al., 2008).

### 4D simulation of the interactions between the catalytic residues

In order to assess the potential impact of the aforementioned differences, we investigated the positional dynamics of the catalytic triad residues during the course of simulation and used the distance distribution profiles of catalytically relevant distances as indicators of the 4D variations.

The distance distribution profiles between N∊2 of H57 and Oγ of S139, as well as between Nδ1 of H57 and Oδ1 of D81, of the template (HCV-1b) and model (HCV-4a) vary widely in both peak value and breadth.

In the template structure (HCV-1b), the distance between Nδ1 of H57 and Oδ1 of D81 exhibits a sharp distribution with a peak value around 3 Å. In the model (HCV-4a), however, the corresponding distance distribution is bimodal, much broader, and distributed around ∼7 and 10 Å (Figure 3(a)). Similar distributions were obtained between Nδ1 of H57 and Oδ2 of D81 (see Supplementary data). Furthermore, the distance distribution between Oγ of S139 and N∊2 of H57 in the template (HCV-1b) shows a peak value at around 4 Å, while the corresponding distribution in the model (HCV-4a) is broader and exhibits a shifted peak value at around 8 Å (Figure 3(b)).

Together, these data indicate that in the model (HCV-4a), H57 spends less time within a probable hydrogen bonding distance to both S139 and D81. Thus, H57 is less likely to act as an efficient general acid–base, as explained previously. This is consistent with the observation that the catalytic activity of HCV-4a NS3 protease is several folds of magnitude less than that of HCV-1b (Franco et al., 2008).

It is important to note that the predicted divergent dynamics behavior (Figure 3(a) and (b)) is totally hidden by the apparent similarity seen in the catalytic site of the rigid structures (Figure 3(c) and (d)). This highlights the importance of utilizing molecular dynamics as a method of future investigations into protease activity.

### T54I mutation may affect the dynamics of His57

**Figure 4.**
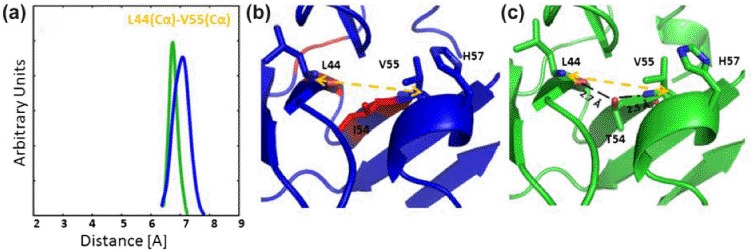
Dynamics behavior near the catalytic H57 in the model (HCV-4a, blue) and the template (HCV-1b, green) proteases (b,c). The distance distribution profiles between Cα atoms of residues L44 and V55 are shown in (a). The effect of T54I mutation in bridging the two adjacent sheets is shown in (b,c).

HCV NS3 protease inhibitors such as Telaprevir and Boceprevir are generally designed to interfere with the catalytic triad (Kwo & Vinayek, 2011; Raney et al., 2010). Precedence for drug resistance due to variability at position T54 has been established (Welsch et al., 2008). In the structure of HCV-1b NS3 protease, T54 is located at the very end of a β-strand (Figure 4(c)), which belongs to an antiparallel β-sheet (Welsch et al., 2008). The hydroxyl group of the T54 side chain is involved in the formation of two H-bonds with the adjacent residues V55 and L44 in the neighboring antiparallel β-strand (Figure 4(c)). The distance between the back-bone H-bond donor and acceptor, L44 and V55, at 4.60 Å is too large to be bridged by a single H-bond (Welsch et al., 2008). The two H-bonds from the T54 residue side chain bridge the two strands and thereby stabilize the local β-sheet conformation (Figure 4(c)).

The sequence of HCV-4a is different from HCV-1b at residue 54 (Figure 1(a) and (d)). The absence of a hydroxyl group in T54I is expected to have an impact on the local β-sheet conformation in HCV-4a protease (Figure 4(b)). To assess the dynamics consequences of that mutation, we used the distance between the Cα atoms of amino acids L44 and V55 as an indicator of the conformational changes. We observed a broader distance distribution (∼50% increase) and an upshift in peak value(∼0.3 Å) between HCV-4a and HCV-1b (Figure 4(a)). Thus, the strands bearing L44 and V55 are expected to spend more time closer to each other in HCV-1b than with HCV-4a.

As H57 is adjacent to V55, any loss of stability in V55, due to the lack of H-bond with I54, may directly impact the dynamics of the catalytic histidine (Figure 4(b)). Thus, the larger oscillation of the V55 bearing strand in HCV-4a when coupled with the H57's large RMSD, points to a histidine that is less capable to perform its catalytic function. This could be an important contribution not only to the higher drug resistivity, but also to the decreased protease activity of HCV-4a compared with HCV-1b as mentioned in Massariol, Zhao, Marquis, Thibeault, and White (2010).

Thirty variability sites exist between the sequences of HCV-1b and HCV-4a (Figure 1(d)) and it is unlikely that T54I, alone, is responsible for the differences in dynamics at the catalytic site. In other words, the genotypic variations express their effects in combination and not as individual mutations. Therefore, our data is relevant only when all these genotypic variations are present in the structure.

Taken together, the data presented here suggest that the structural dynamics within the catalytic triad are significantly different between HCV-1b and HCV-4a NS3 proteases. The effect of sequence variability in the HCV-4a protease seems to extend beyond the rigid structure to render the molecular dynamics less accommodating to natural substrates and, possibly, potential drugs. It is evident that there are numerous details underpinning the causes of the genotype-based drug resistance. However, the changes in the dynamic behavior at the catalytic triad, between drug responsive and drug resistant HCV genotypes, provide a new avenue of inquiry with suggestive results. The final confirmation awaits the crystal structures of HCV-4a protease, with and without bound drugs, along with computationally intensive analyses of the protease–drug complexes, and the interplay between sequence variability, dynamic behavior, and drug binding.

## Supplementary material

The supplementary material for this paper is available online at http://dx.doi.10.1080/07391102.2013.800001.

## Abbreviations

HCV: hepatitis C virus
NS: nonstructural
RMSD: root mean square deviation
Cα: alpha carbon
